# Synergic effect of arsenic exposure related methylation changes in three cohorts exposed to levels of this toxicant

**DOI:** 10.1007/s00420-025-02147-6

**Published:** 2025-05-26

**Authors:** Katarzyna Ewa Sokolowska, Jacek Antoniewski, Marta Sobalska-Kwapis, Dominik Strapagiel, Wojciech Marciniak, Jan Lubiński, Tomasz Kazimierz Wojdacz

**Affiliations:** 1 Independent Clinical Epigenetics Laboratory, Pomeranian Medical University, Unii Lubelskiej 1, Szczecin, 71-252 Poland; 2 Biobank Laboratory, Departament of Oncobiology and Epigenetics, Faculty of Biology and Environmental Protection, University of Lodz, Pomorska 139 St, Lodz, 90-235 Poland; 3 Department of Genetics and Pathology, Pomeranian Medical University, Szczecin, Poland; 4 Read-Gene SA, Grzepnica, Poland

**Keywords:** DNA methylation, Arsenic, As, Blood, Urine

## Abstract

**Purpose:**

The results of studies assessing impact of arsenic exposure on methylome are to large extent inconsistent. To contribute to understanding of effect of arsenic exposure on methylome of the exposed cells, we assess the impact of low-level arsenic exposure on methylome of blood cells in three cohorts of exposed individuals.

**Methods:**

The Infinium MethylationEPIC array (Illumina Inc.) was used for genome-wide methylation profiling and robust linear regression to identify arsenic-related methylation changes in blood cells from healthy individuals with a 12-year cancer-free follow-up and breast cancer patients, sampled on average 4.29 years before diagnosis, as well as methylomics data from cord blood samples of Biomarkers of Exposure to Arsenic cohort.

**Results:**

Our analysis identified a 2,453 arsenic-associated methylation changes in blood from healthy individuals, 9,662 in breast cancer patients and 6,745 in cord blood samples. Similarly to previous studies methylation changes that we identified in each cohort, overlapped only to some extent. However, molecular processes linked to identified methylation changes were very similar in each of the cohorts. And included pathways that could be clearly associated with the adverse effects of arsenic exposure and specifically cancer in the cohort of cancer patients. Moreover, the genomic regions harboring identified in each cohort methylation changes were similar and predominantly included regions participating in regulation of gene transcription.

**Conclusion:**

Overall, our findings show that specificity of arsenic related methylation changes is low but the impact of these changes on cell physiology is very similar across three cohorts we studded.

**Supplementary Information:**

The online version contains supplementary material available at 10.1007/s00420-025-02147-6.

## Introduction

Arsenic (As), naturally occurring toxic metalloid, frequently present in soil and consequently crops grown in the regions with high natural As contamination. Chronic exposure to arsenic, particularly through contaminated drinking water, has been associated with a range of adverse health effects, such as cardiovascular diseases, neurotoxicity, and skin disorders (Pierce et al. 2011; Tellez-Plaza et al. 2018; Thakur et al. 2021). Moreover, because exposure to arsenic has been linked to increased risk of skin, bladder, lungs, kidney, liver and possibly prostate cancers (IARC Working Group on the Evaluation of Carcinogenic Risks to Humans 2012), International Agency for Research on Cancer (IARC) and the World Health Organization (WHO) classified arsenic as class 1 human carcinogen. We have also shown that women from the Pomeranian region of Poland with elevated blood arsenic levels had a 13-fold increased risk of developing breast cancer (hazard ratio [HR] = 13.2; 95% confidence interval [CI] 4.02-43 (Marciniak et al. 2020). Countries most severely affected by exposure to arsenic naturally occurring in drinking water include Bangladesh, Cambodia, China, India, Myanmar, Nepal, Pakistan, Taiwan, Argentina, and Vietnam (Ravenscroft et al. 2009).

Exposure to arsenic from contaminated water, primarily in its inorganic form, in a number of studies has been linked to alterations in the methylome of blood cells as reviewed in (EFSA Panel on Contaminants in the Food Chain (CONTAM) et al. 2024). These methylation changes are likely to disrupt cellular pathways involved in physiological processes that have been associated with the arsenic exposure such as cell proliferation, induce cytotoxicity, oxidative stress and genomic instability (Zhou and Xi 2018). However, there is general lack of consistency between studies reporting arsenic exposure related methylation changes with some studies identifying a large number of methylation changes in blood of exposed individuals (Cardenas et al. 2015a; Rojas et al. 2015; Lumour-Mensah and Lemos 2024), others only a few (Green et al. 2016; Kaushal et al. 2017; Argos et al. 2018; Guo et al. 2018; Demanelis et al. 2019; Bozack et al. 2020; Xiao et al. 2022; Wei et al. 2022, 2024), or lack of arsenic-related methylome aberrations after correction for multiple testing (Koestler et al. 2013; Kile et al. 2014; Seow et al. 2014; Broberg et al. 2014; Cardenas et al. 2015b; Ameer et al. 2017; Gliga et al. 2018). The discrepancies between studies are not surprising given significant differences between studies regarding study design and measurements of arsenic exposure. Nevertheless, there is no doubt that adverse effects of arsenic exposure are mediated by exposure related methylation changes.

In this study, we aimed to identify arsenic-associated DNA methylation changes and assess their involvement in molecular mechanisms underlying arsenic-induced health effects.

We identified methylation changes associated with arsenic levels in three cohorts of exposed individuals. These changes uniformly across those cohorts affected very specific regulatory regions of the genome and were associated with the molecular processes and cellular pathways that are in line with observed adverse health effects of arsenic exposure including its carcinogenic effect.

## Methods

### Study participants characteristics

The characteristics of the three cohorts of women included in our study are summarized in Table 1. Overall, the study included 56 healthy with 12-years cancer free follow-up and 34 women diagnosed with breast cancer with blood samples collected 4.29 years prior to cancer diagnosis from the Pomeranian region of Poland. As well as data from public repositories from 38 mother-infant pairs from the larger Biomarkers of Exposure to Arsenic (BEAR) cohort from Mexico (GSE62924) (Rojas et al. 2015).

**Table 1:** Characteristics of study participants

	Healthy women	Women with BC	Mother-infant pairs
n	56	34	38
Age (years)			
Mean Median Range SD	52.75 52 40–69 7.69	54.94 53 35–77 10.62	23.42 20.50 18–39 6.06
Total arsenic in blood (µg/L)			
Mean Median Range SD	2.06 2.09 0.22–5.77 1.41	1.39 1.33 0.65–4.31 0.66	- - - -
Total urinary arsenic SG normalized (µg/L)			
Mean Median Range SD	- - - -	- - - -	67.40 30.19 6.18-319.74 78.90

## DNA extraction and as level measurement

DNA from whole blood samples was extracted using the salting-out method (Lahiri and Schnabel 1993) and from cord blood samples in the mother-infant pairs using the PaxGene Blood DNA kit (Qiagen, Valencia, CA) (Rojas et al. 2015). In blood samples from our institution, total blood arsenic, attributed primarily to exposure from food, was measured using inductively coupled plasma mass spectrometry (ICP-MS) with an Elan Dynamic Reaction Cell-e instrument (PerkinElmer, Waltham, MA), following protocol outlined in (Marciniak et al. 2020). In samples from Biomarkers of Exposure to Arsenic (BEAR) cohort, total urinary arsenic levels were determined as the standard gravity-adjusted sum of the major arsenic species including inorganic arsenic (iAs), monomethylarsonate (MMA) and dimethylarsinate (DMA). The concentrations of each of the arsenic species were measured using hydride generation-atomic absorption spectrometry (HG-AAS) with cryotrapping (Devesa et al. 2004; Hernandez-Zavala et al. 2009).

## Genome-wide methylation data processing

Genome-wide DNA methylation profiling was performed using the Infinium MethylationEPIC array (Illumina Inc.) following the manufacturer protocol. Data preprocessing and quality assessment were performed using ChAMP R package (Morris et al. 2014; Tian et al. 2017). To minimize potential bias from type-2 probes, a beta-mixture quantile intra-sample normalization procedure (BMIQ) was applied, as previously described (Teschendorff et al. 2013). After the quality assessment methylation level data for 732,679 CpG sites were included in the analysis. We were not able to process methylation profiling data from Biomarkers of Exposure to Arsenic (BEAR) cohort with the same workflow because authors deposited these data as beta-values, normalized using a quantile-based methodology in text file. This dataset consisted of methylation levels for 484,667 CpG sites of which 412,265 CpG sites was used in our analysis after removing probes not targeting CpGs, probes with SNPs, probes that align to multiple locations and probes located on X and Y chromosomes.

## Identification of CpG sites with methylation levels associated with as exposure

We used robust linear models implemented in R limma package with empirical Bayes smoothing of standard errors to identify CpG sites with methylation levels associated with arsenic exposure (Ritchie et al. 2015). To meet linear regression model assumptions, Beta-values ranging from 0 (no methylation) to 1 (100% methylation) were transformed to M-values according to equation: M-value = ln[Beta-value/(1-Beta-value)] (Du et al. 2010). Models were adjusted for age and blood cell type proportion estimates, which were calculated using outperforming robust partial correlation method implemented in EpiDISH R package (Houseman et al. 2012; Newman et al. 2015; Teschendorff et al. 2017) with “centDHSbloodDMC.m” dataset (Teschendorff et al. 2017) as reference. For each dataset we individually assessed the limits of Benjamini-Hochberg False Discovery Rate correction for multiple testing (FDR corrected p-value; q-value) and cutoff of q-value ≤ 10e-8 was used in the analysis of data from healthy women from our institution and q-value ≤ 0.05 for data from Biomarkers of Exposure to Arsenic (BEAR) cohort as well as data from women diagnosed with breast cancer from our institution. All processing of DNA methylation array data and analyses were conducted in R 4.2.2.

## Genome regions enrichment analysis

To assess whether identified methylation changes occupy specific genomic regions, we analyzed the distribution of loci harboring these changes relative to regions marked by histones with specific modification in 11 cell types including: monocytes, neutrophils, B cells, natural killers, mononuclear cells, and seven types of T cells (samples id: E029, E030, E032, E034, E037, E039, E043, E044, E046, E047, E048) from Roadmap Epigenomics Core 15-state Model (Roadmap Epigenomics Consortium et al. 2015), using Locus Overlap Analysis (LOLA) (Sheffield and Bock 2016). All CpGs that undergo QC during processing were used as a background in these analyses. As significantly enriched results we considered those with FDR corrected p-value ≤ 0.05 (Fisher exact test).

### Gene ontology term enrichment and fold change analyses

To establish which biological pathways may be affected by the identified methylation changes, we performed Gene Set Enrichment Analysis (GSEA) using “GENE2FUNC” function in FUMA (Watanabe et al. 2017). The top 10 ontology terms were analyzed within two databases: “Hallmark gene sets” and “GO biological processes”.

## Results

Our initial analysis of methylation profiling data from blood cells of healthy women showed that methylation levels at 2,453 CpG sites (1,794 positively and 659 negatively; Supplementary Material 1 Table S1) (q-value ≤ 10e-8) were associated with the blood arsenic levels. To approximate the physiological pathways affected by the identified methylation changes we annotated these changes to genes and performed GSEA using FUMA platform (Supplementary Material 2 Table S1). We based this analysis on “Hallmark gene sets” and “GO biological processes” ontology terms databases. The “Hallmark gene sets” based GSEA analysis linked three ontology terms associated to identified in this cohort methylation changes. Two of those terms were: HALLMARK_MITOTIC_SPINDLE and HALLMARK_G2M_CHECKPOINT. These results are in line with previews reports indicating that arsenic exposure disrupts cell cycle (Wu et al. 2013; States 2015; Ganapathy et al. 2019). Moreover, we and others have shown that arsenic exposure is associated with alterations in chromatin structure (Riedmann et al. 2015; Engström et al. 2017). Similarly, majority of the ontology terms linked to methylation changes identified for this cohort in “GO biological processes” based GSEA analysis, were cell cycle related terms including: GOBP_MITOTIC_CELL_CYCLE_PROCESS, GOBP_MITOTIC_CELL_CYCLE, GOBP_CELL_CYCLE_PROCESS, GOBP_MITOTIC_CELL_CYCLE_PHASE_TRANSITION, GOBP_CHROMATIN_ORGANIZATION, GOBP_CELL_CYCLE_PHASE_TRANSITION, and GOBP_REGULATION_OF_MITOTIC_CELL_CYCLE_PHASE_TRANSITION.

The third ontology term in “Hallmark gene sets” based GSEA analysis was HALLMARK_PROTEIN_SECRETION and previous studies have demonstrated that sub-chronic arsenic exposure enhances the secretion of pro-inflammatory cytokines (Zhang et al. 2016).

Also, “GO biological processes” based GSEA analysis identified GOBP_CELLULAR_RESPONSE_TO_DNA_DAMAGE_STIMULUS and arsenic related DNA damage is well established mechanism of arsenic-related carcinogenesis (Martinez et al. 2011).

**Fig. 1:**
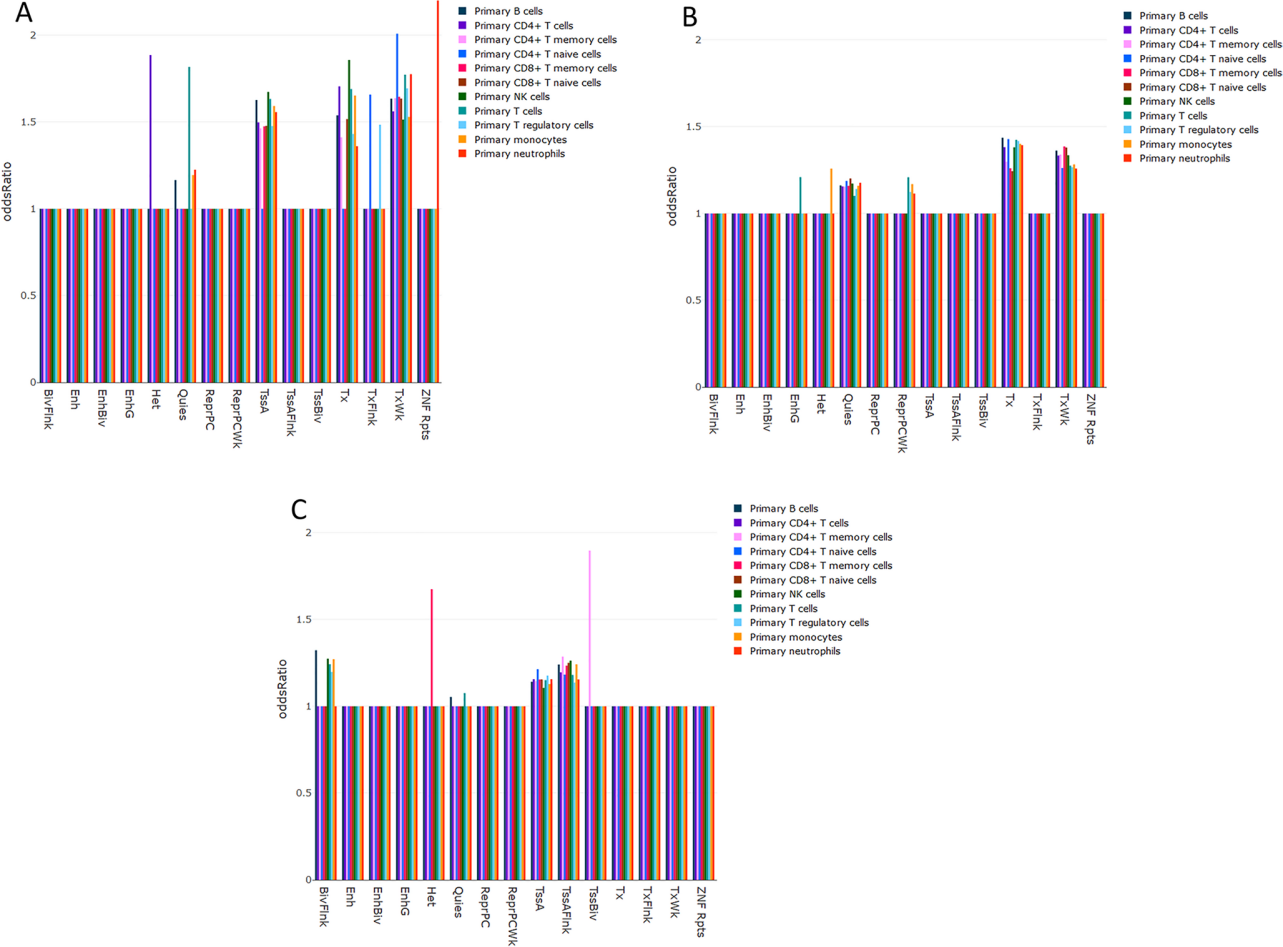
Results of enrichment analysis of identified methylation changes with genomic regular regions. Histograms illustrate the results of enrichment analysis of CpG sites identified in each of the cohorts in the study in regions marker with specific histone modifications according to Core 15-state model in 11 types of blood cells. Panel A – results for blood of healthy women with the enrichment of arsenic exposure associated methylation changes in transcription start sites of actively transcribed genes (TssA), gene bodies of actively transcribed genes with strong transcription (Tx), and gene bodies of actively transcribed genes with weak transcription (TxWk). Panel B – results for cord blood with enrichment observed also in Tx, and TxWk and inactive regions of the genome (Quies). Panel C – for blood of women with breast cancer with enrichment observed in TssA and regions flanking TSS of actively transcribed genes (TssAFlnk)

Most interestingly however, the analysis of enrichment of CpG sites associated with blood arsenic in this cohort with the genomic regions marked with specific histone marks, showed that these CpG sites are enriched in regions marked with histones characteristic for transcription start sites (TssA) and gene bodies (Tx and TxWk) of actively transcribed genes (Fig. 1A). These results suggest that arsenic exposure interferes with cellular processes by disrupting methylation levels at CpG sites in promoters and gene bodies.

Due to the limitations of our study we attempted to validate our findings using data from independent study. Unfortunately, we did not identify publicly available methylomics data from the similar to our cohort. However, we were able to access genome-wide methylation profiling data from 38 cord blood samples accompanied with the measurement of total urinary arsenic adjusted for standard gravity. A linear regression-based analysis, identical to the one we performed with data from healthy women, identified only one CpG site that reached FDR adjusted significance at q-value ≤ 10e-8 in these samples. However, when controlling for the FDR at 5% (q-value < 0.05), we found 6,745 CpG sites (3,072 positively and 3,673 negatively; Supplementary Material 1 Table S2) associated with total urinary arsenic levels normalized for standard gravity (µg/L). The analysis of association of this subset of CpG sites with histone marks, again showed that regions harboring identified here methylation aberrations are enriched in regions marked with histones characteristic for gene bodies of actively transcribed genes (Tx and TxWk) (Fig. 1B). Also, again here, one of top ontology terms linked in “Hallmark gene sets” based GSEA analyses to methylation changes identified in this cohort was HALLMARK_MITOTIC_SPINDLE and this process we not only identified in the analysis of data from healthy women cohort but also is a key process for majority of terms identified in “GO biological processes” based GSEA analysis performed for this cohort (Supplementary Material 2 Table S2). Additionally, “GO biological processes” based GSEA analysis in this cohort identified several terms related to neurogenesis, including: GOBP_NEUROGENESIS, GOBP_GENERATION_OF_NEURONS, GOBP_NEURON_DEVELOPMENT, and neurotoxicity is one of the main adverse effects of the arsenic exposure (Tyler and Allan 2014). Interestingly, also among terms identified in “Hallmark gene sets” based GSEA analysis were HALLMARK_WNT_BETA-CATENIN_SIGNALING and HALLMARK_ESTROGEN_RESPONSE_EARLY. In the study utilizing P19 mouse embryonic stem cells, arsenic was shown to disrupt cell morphogenesis by repressing Wnt/β-catenin signaling (Hong and Bain 2012), and arsenic exposure was also previously shown to affect the expression of estrogen-related genes (Davey et al. 2007; Che et al. 2019).

Lastly, we analyzed the association of blood arsenic levels with genome-wide methylation patterns in blood cells from women for which we have previously shown 13-fold increase in risk of breast cancer incidence associated with arsenic exposure (Marciniak et al. 2020). This analysis at FDR q-value ≤ 0.05 identified 9,662 CpG sites (4,838 positively and 4,824 negatively; Supplementary Material 1 Table S3) significantly associated with blood arsenic levels.

Similarly to previous analyses, the majority of these methylation changes were enriched in regions marked by histones characteristic for transcription start site (TssA) and also regions flanking TSS (TssAFlnk) of actively transcribed genes (Fig. 1C).

Also consistently with two previous analyses, the GSEA based on this subset of CpG sites (Supplementary Material 2 Table S3) linked identified here methylation changes with cellular processes previously shown to be associated with adverse arsenic exposure. Specifically, among 10 top hits in “Hallmark gene sets” based GSEA analysis, three ontology terms identified here were also present in the analysis of previous two cohorts and included: HALLMARK_MITOTIC_SPINDLE, HALLMARK_WNT_BETA_CATENIN_SIGNALING and HALLMARK_ESTROGEN_RESPONSE_EARLY. Moreover, again similarly to the GSEA analyses of other cohorts in our study, terms related to cell cycle, morphogenesis and neuronal development, constituted majority of the ontology terms identified in both “Hallmark gene sets” and “GO biological processes” based GSEA analyses performed for methylation changes identified in this cohort. Interestingly, also among the top hits in this analysis were terms related to well-known oncogenic processes including HALLMARK_EPITHELIAL_MESENCHYMAL_TRANSITION, HALLMARK_KRAS_SIGNALING_UP and HALLMARK_APOPTOSIS what may indicate direct carcinogenic effect of arsenic exposure.

## Discussion

Previous Epigenome-Wide Association Studies (EWAS) demonstrated that arsenic exposure can significantly influence blood cells methylome (Liu et al. 2014; Argos et al. 2015, 2018; Ameer et al. 2017; Demanelis et al. 2019; Bozack et al. 2020, 2021; Domingo-Relloso et al. 2022a; Wang et al. 2023) and arsenic induced methylation changes have been associated with a number of adverse health effects (Seow et al. 2014; Guo et al. 2018; Domingo-Relloso et al. 2022b; Xiao et al. 2022; Wei et al. 2022, 2024; Gao et al. 2023). However, there are large discrepancies between studies reporting arsenic related methylation changes. Some of these studies report only a few arsenic exposure related methylation aberrations (Green et al. 2016; Kaushal et al. 2017; Argos et al. 2018; Guo et al. 2018; Demanelis et al. 2019; Bozack et al. 2020; Xiao et al. 2022; Wei et al. 2022, 2024), others thousands (Cardenas et al. 2015a; Rojas et al. 2015; Lumour-Mensah and Lemos 2024) and some report no statistically significant (especially after correction for multiple testing) arsenic exposure related methylation changes (Koestler et al. 2013; Kile et al. 2014; Seow et al. 2014; Broberg et al. 2014; Cardenas et al. 2015b; Ameer et al. 2017; Gliga et al. 2018). Also, our review of published studies showed that the majority of the studies do not report methylation changes at similar loci (Liu et al. 2014; Kile et al. 2014; Seow et al. 2014; Green et al. 2016; Gliga et al. 2018; Demanelis et al. 2019; Xiao et al. 2022; Wang et al. 2023). These discrepancies are not surprising and are most likely attributed to differences between studded cohorts as well as arsenic exposure levels, different data analysis approaches and methodologies of arsenic exposure assessment. Overall, there is no doubt that arsenic affects methylomes of the exposed cells, however, no specific genomic regions harboring arsenic exposure related methylation changes have been identified.

In our study we also found different number of arsenic-related methylation changes across the three cohorts analyzed. However, the genomic regions harboring changes we identified were almost identical in each of the three studded cohorts. Those regions were gene promoters and gene body regions of actively transcribed genes, both of which have been shown to be involved in gene expression regulation (Yang et al. 2014).

Some of the previous EWAS studies found enrichment of arsenic exposure related methylation changes in promoters (Kaushal et al. 2017; Guo et al. 2018; Wang et al. 2023) and gene bodies of active genes (Rojas et al. 2015; Gao et al. 2023) but there also studies that reported depletion of arsenic related methylation changes in promoters (Rojas et al. 2015; Demanelis et al. 2019). In our study we consistently identified similar genomic regions in three independent cohorts.

Also, the GSEA analyses that we performed for the subset of methylation changes identified in each cohort, consistently linked these changes to the molecular processes that have previously in various studies, been associated with arsenic exposure such as cell cycle regulation, morphogenesis, and neurogenesis (Liu et al. 2014; Broberg et al. 2014; Ameer et al. 2017; Kaushal et al. 2017; Gliga et al. 2018; Kim et al. 2022). These results in the context of previous studies again confirm that despite that arsenic does not seem to affect methylation of specific genes, overall arsenic related methylation changes affect loci involved in very similar molecular processes.

A limitation of this study is the relatively small sample size, which may restrict the generalizability of our findings. To address this, we performed validation using an independent cohort. We found that the only available methylation data from public repositories were from a subset of individuals in the BEAR cohort (mother-infant pairs), previously described in (Rojas et al. 2015). While this cohort is not an ideal match for our study population, it provided an opportunity to validate our findings.

Despite these limitations, this study offers valuable insights into the effects of arsenic exposure. However, further, more detailed research is necessary for a more comprehensive understanding of the phenomenon.

### Conclusions

Previous studies have without doubt shown that adverse health effects of arsenic exposure are, to a large extent, attributed to influence of this toxic metalloid on methylome of exposed cells. Our results presented here add to understanding of molecular mechanisms of arsenic toxicity and suggest that arsenic induces methylation aberrations at the transcription start site and gene body of transcriptional active genes and through that, disrupts cellular processes that have previously been shown to be involved in arsenic toxicity both in blood and cord blood cells.

## Electronic supplementary material

Below is the link to the electronic supplementary material.

Supplementary Material 1

Supplementary Material 2

Supplementary Material 3

## Data Availability

The datasets generated and/or analyzed during the current study are available in the Gene Expression Omnibus repository, GSE283951 – our data and GSE62924.

## Abbreviations

BEAR: Biomarkers of Exposure to Arsenic
BMIQ: Beta-mixture quantile intra-sample normalization procedure
CI: Confidence interval
DMA: Dimethylarsinate
EWAS: Epigenome-Wide Association Studies
FDR: False Discovery Rate
GSEA: Gene Set Enrichment Analysis
HG-AAS: Hydride generation-atomic absorption spectrometry
HR: Hazard ratio
IARC: International Agency for Research on Cancer
iAs: Inorganic arsenic
ICP-MS: Inductively coupled plasma mass spectrometry
LOLA: Locus Overlap Analysis
MMA: Monomethylarsonate
TssA: Transcription start sites of actively transcribed genes
TssAFlnk: Regions flanking TSS of actively transcribed genes
Tx: Gene bodies of actively transcribed genes (strong transcription)
TxWk: Gene bodies of actively transcribed genes (weak transcription)
WHO: World Health Organization
