# Supplementary material for: Synergic effect of arsenic exposure related methylation changes in three cohorts exposed to levels of this toxicant
Source: Int Arch Occup Environ Health. 2025 May 26;98(6):515–23. doi: 10.1007/s00420-025-02147-6 (PMC12331777; doi:10.1007/s00420-025-02147-6)
Supplement: Supplementary file 3 — Supplementary Material 3 [file 420_2025_2147_MOESM3_ESM.docx]

**Additional Files**

**Additional File 1 (Additional_File_1.xlsx)**

**Table S1**: CpG sites identified for healthy Polish women

**Table S2**: CpG sites identified for Mexican mother-infant pairs

**Table S3**: CpG sites identified for Polish woman with breast cancer

**Additional File 2 (Additional_File_2.xlsx)**

**Table S1**: GSEA results for healthy Polish women, showing the top 10 ontology terms from the "Hallmark gene sets" database and the top 10 ontology terms from the "GO biological processes" database. The analysis used genes annotated to 2,453 CpG sites as input.

**Table S2**: GSEA results for mother-infant pairs, showing the top 10 ontology terms from the "Hallmark gene sets" database and the top 10 ontology terms from the "GO biological processes" database. The analysis used genes annotated to 6,745 CpG sites as input.

**Table S3**: GSEA results for Polish women diagnosed with breast cancer (BC), showing the top 10 ontology terms from the "Hallmark gene sets" database and the top 10 ontology terms from the "GO biological processes" database. The analysis used genes annotated to 9,662 CpG sites as input.
